# Supplementary figures and images for: A Novel Terrestrial Rabies Virus Lineage Occurring in South America: Origin, Diversification, and Evidence of Contact between Wild and Domestic Cycles
Source: Viruses. 2021 Dec 11;13(12):2484. doi: 10.3390/v13122484 (PMC8707302; doi:10.3390/v13122484)

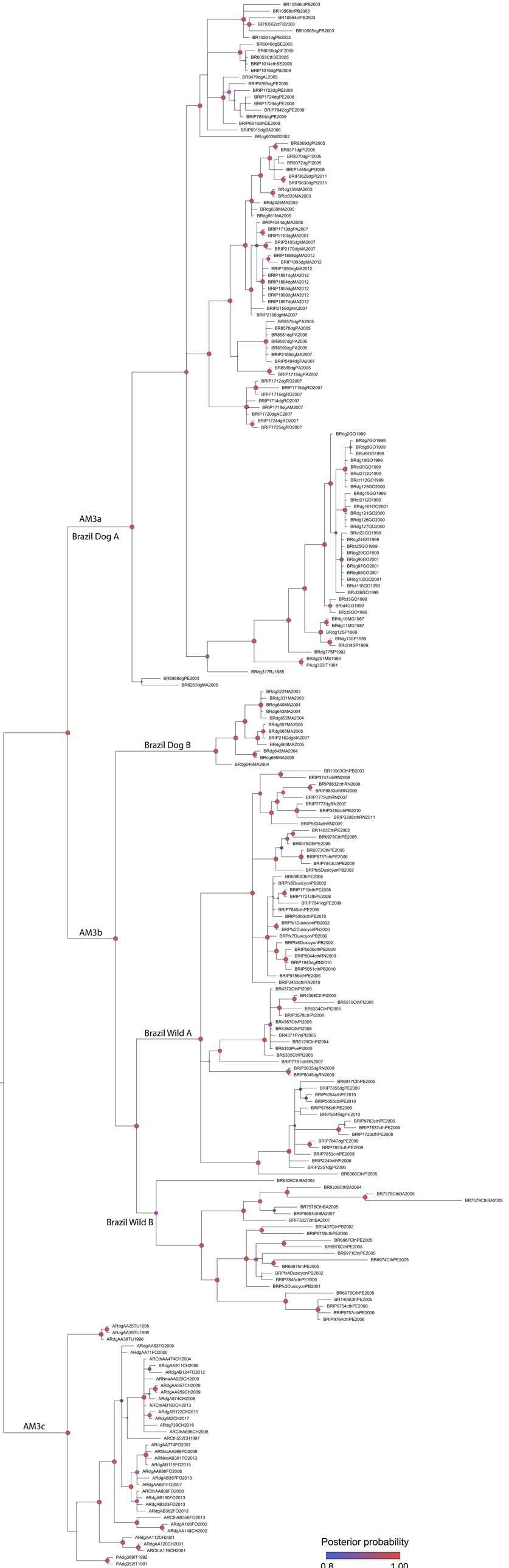

Supplement: Supplementary file 1 [file viruses-13-02484-s001.zip › Supplementary Figure S1.pdf]

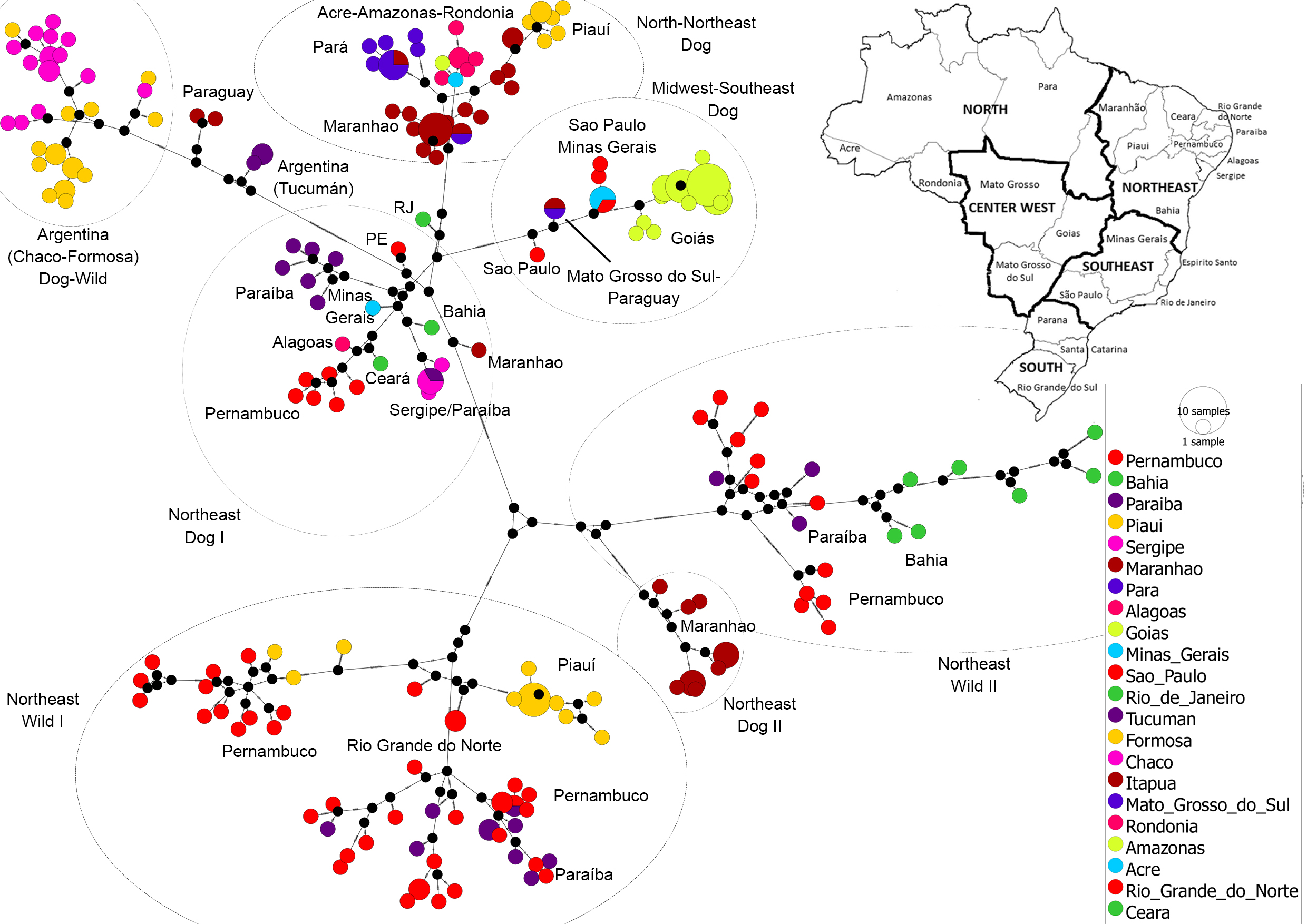

Supplement: Supplementary file 1 [file viruses-13-02484-s001.zip › Supplementary Figure S2.png]

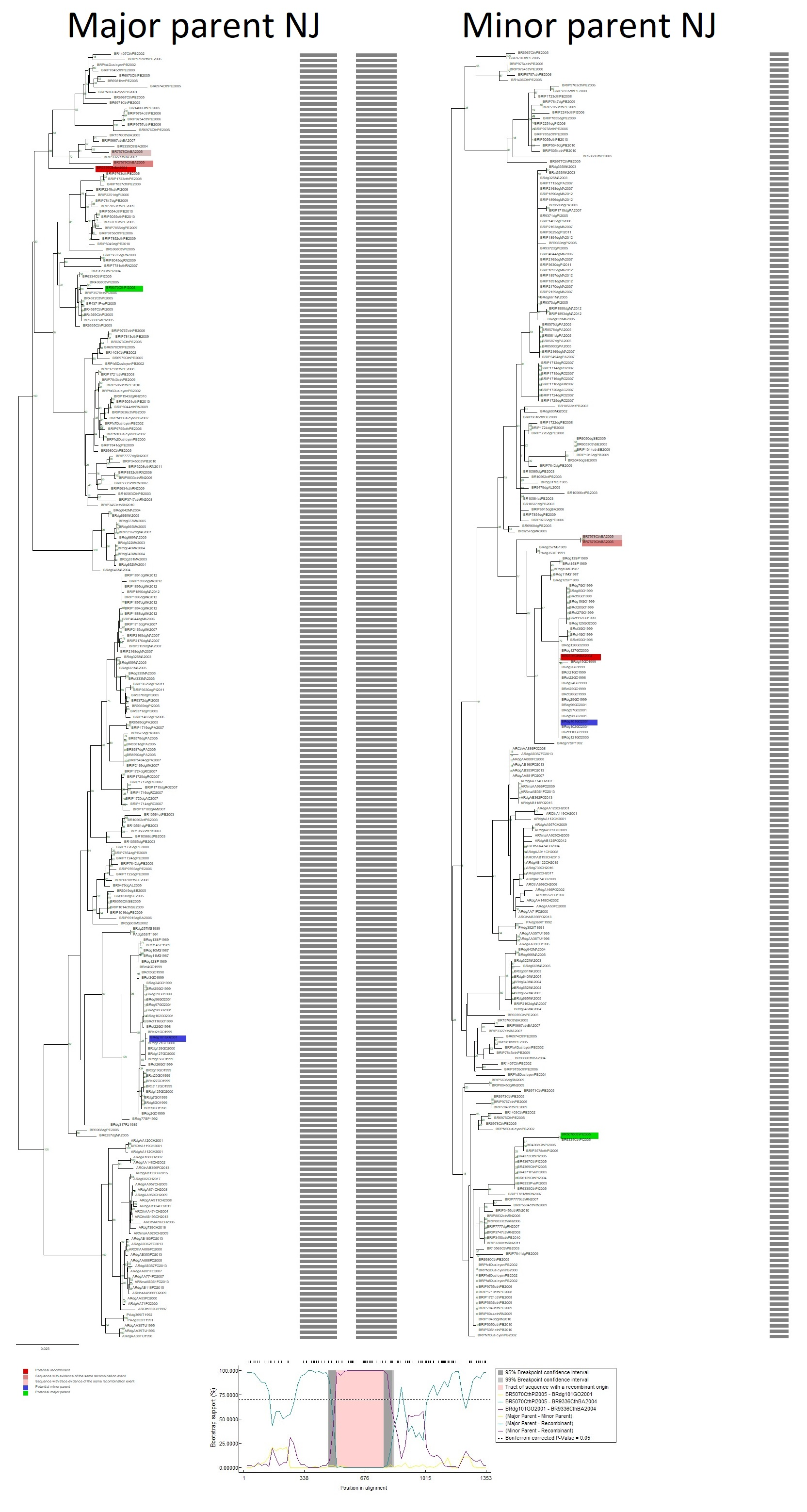

Supplement: Supplementary file 1 [file viruses-13-02484-s001.zip › Supplementary Figure S3.png]
